# Elevation in sphingolipid upon SARS-CoV-2 infection: possible implications for COVID-19 pathology

**DOI:** 10.26508/lsa.202101168

**Published:** 2021-11-11

**Authors:** Einat B Vitner, Roy Avraham, Boaz Politi, Sharon Melamed, Tomer Israely

**Affiliations:** Departments of Infectious Diseases, Israel Institute for Biological Research, Ness-Ziona, Israel

## Abstract

Understanding pathways that might impact coronavirus disease 2019 (COVID-19) manifestations and disease outcomes is necessary for better disease management and for therapeutic development. Here, we analyzed alterations in sphingolipid (SL) levels upon infection with severe acute respiratory syndrome coronavirus 2 (SARS-CoV-2). SARS-CoV-2 infection induced elevation of SL levels in both cells and sera of infected mice. A significant increase in glycosphingolipid levels was induced early post SARS-CoV-2 infection, which was essential for viral replication. This elevation could be reversed by treatment with glucosylceramide synthase inhibitors. Levels of sphinganine, sphingosine, GA1, and GM3 were significantly increased in both cells and the murine model upon SARS-CoV-2 infection. The potential involvement of SLs in COVID-19 pathology is discussed.

## Abstract (precis)

SARS-CoV-2 infection alters the levels of sphingolipids early post infection. This phenomenon is reflected by increased levels of sphingolipids, including gangliosides, in infected cells, as well as in serum in a SARS-CoV-2 murine model.

## Introduction

In December 2019, the novel coronavirus severe acute respiratory syndrome coronavirus-2 (SARS-CoV-2) was identified as the causative agent of a cluster of acute atypical pneumonia cases in the city of Wuhan, China (Zhou et al, 2020). In February 2020, the World Health Organization named the disease COVID-19 (Yang & Wang, 2020).

COVID-19 primarily manifests as a respiratory tract infection causing hypoxemic respiratory failure. However, there is an enormous amount of data demonstrating that it may involve multiple organ systems, including the nervous, cardiovascular, respiratory, gastrointestinal, renal, hematopoietic, and immune systems (Erdinc et al, 2021). Understanding pathological pathways involved in COVID-19 manifestations might reveal new approaches for therapeutic strategies and disease management.

Sphingolipids (SLs) are a major class of eukaryotic cell membrane constituents. In addition to playing a structural role, some SLs are bioactive and control vital biological functions by regulating signal transduction pathways involved in several processes. Some bioactive SLs are implicated in pathological processes, including inflammation-associated illnesses such as atherosclerosis, rheumatoid arthritis, inflammatory bowel disease, type II diabetes, obesity, cancer, and neurological and neurodegenerative diseases (Ogretmen, 2018; D’Angelo et al, 2019; Hussain et al, 2019; Gomez-Larrauri et al, 2020). Furthermore, SLs play an important role in the control of virus replication and the innate immune response (Schneider-Schaulies & Schneider-Schaulies, 2013; Schneider-Schaulies & Schneider-Schaulies, 2015; Bezgovsek et al, 2018; Yager & Konan, 2019; Melamed et al, 2020; Vitner, 2020).

**Figure S1.**
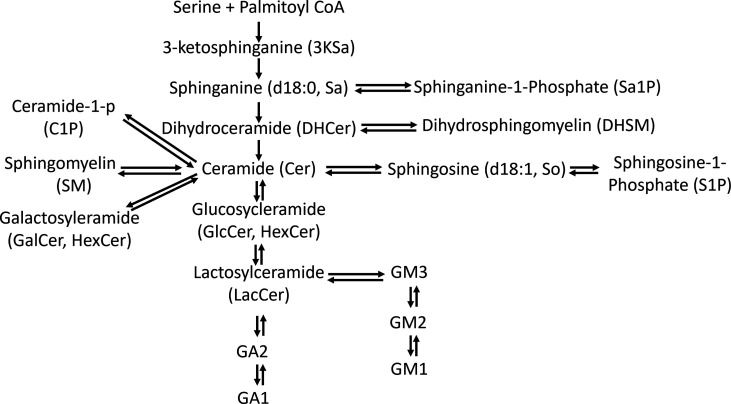
The SL de novo synthesis pathway begins with the condensation of serine and palmitoyl-CoA to form 3-ketosphinganine (3KSa), a reaction catalyzed by serine palmitoyltransferase (SPT). Then, 3-ketosphinganine reductase (KSR) mediates the reduction of 3KSa to sphinganine d18:0 (Sa), which is transformed into dihydroceramide (DHCer) through acylation by ceramide synthases (CerSs). Ceramide (Cer) is formed through the introduction of an (E)-4 double bond into dhCer by dihydroceramide desaturase (Des1). Once formed, Cer can be degraded through the catabolic route, which involves N-deacylation to sphingosine (So, sphinganine d18:1) by ceramidases, further phosphorylation to sphingosine 1-phosphate (S1P) and final irreversible cleavage by S1P lyase (Zheng et al, 2006). Cer is the core structure of all complex SLs, which are generated by attachment of different polar head groups at the primary alcohol group (C1–OH) of a ceramide molecule. Depending on the type of polar group, two major classes have been defined: phosphosphingolipids (such as ceramide-1-p [C1P] and sphingomyelin [SM]) and glycosphingolipids (GSLs) (Mencarelli & Martinez–Martinez, 2013). GSLs are a heterogeneous group of membrane lipids formed by a Cer backbone covalently linked to a glycan moiety. Because these molecules are produced from Cer precursors, they may also have differences in their acyl chain composition, revealing an additional layer of variation. GSLs are divided broadly into two categories: glucosphingolipids and galactosphingolipids. Glucosphingolipids depend initially on the enzyme glucosylceramide (GlcCer) synthase (GCS), which attaches glucose as the first residue to the C1 hydroxyl position (D’Angelo et al, 2013).

Bioactive SLs are regulated by various enzymes and fluxes of different metabolites, with ∼40 enzymes involved in their metabolism in mammals (Hannun & Obeid, 2018) (see Fig S1 for the SL synthesis pathway). Glycosphingolipids (GSLs) are a heterogeneous group of membrane lipids formed by a Cer backbone covalently linked to a glycan moiety. Glucosphingolipids depend initially on the enzyme glucosylceramide (GlcCer) synthase (GCS), which attaches glucose as the first residue to the C1 hydroxyl position (D’Angelo et al, 2013).

**Figure 1.**
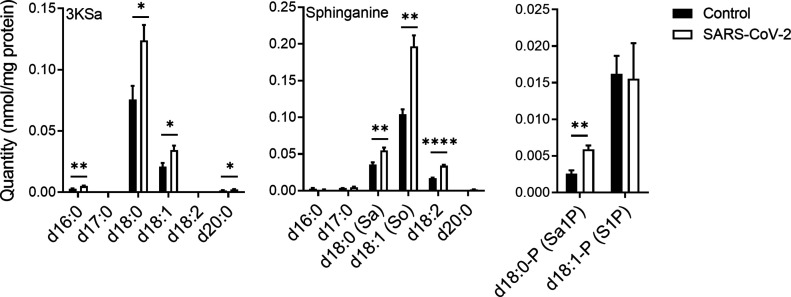
SARS-CoV-2 infection induces elevation of sphingoid base levels in vitro. Vero E6 cells were infected with SARS-CoV-2 at a MOI of 5, and SL levels were quantified at 3 hpi by LC–MS. 3KSa, 3-ketosphinganine; Sa, sphinganine; Sa1P, sphinganine-1-phosphate; S1P, sphingosine-1-phosphate. Data are the means of four biological replicates ± SEMs. Statistical analysis was performed using a two-tailed unpaired *t* test. *P*-values are indicated by asterisks, as follows: *P < 0.05, **P < 0.01, and ****P < 0.0001. Differences with a *P*-value of 0.05 or less were considered significant. Graphs were generated using GraphPad Prism software version 8.4.3. Complete dataset in Table S1.

**Figure 2.**
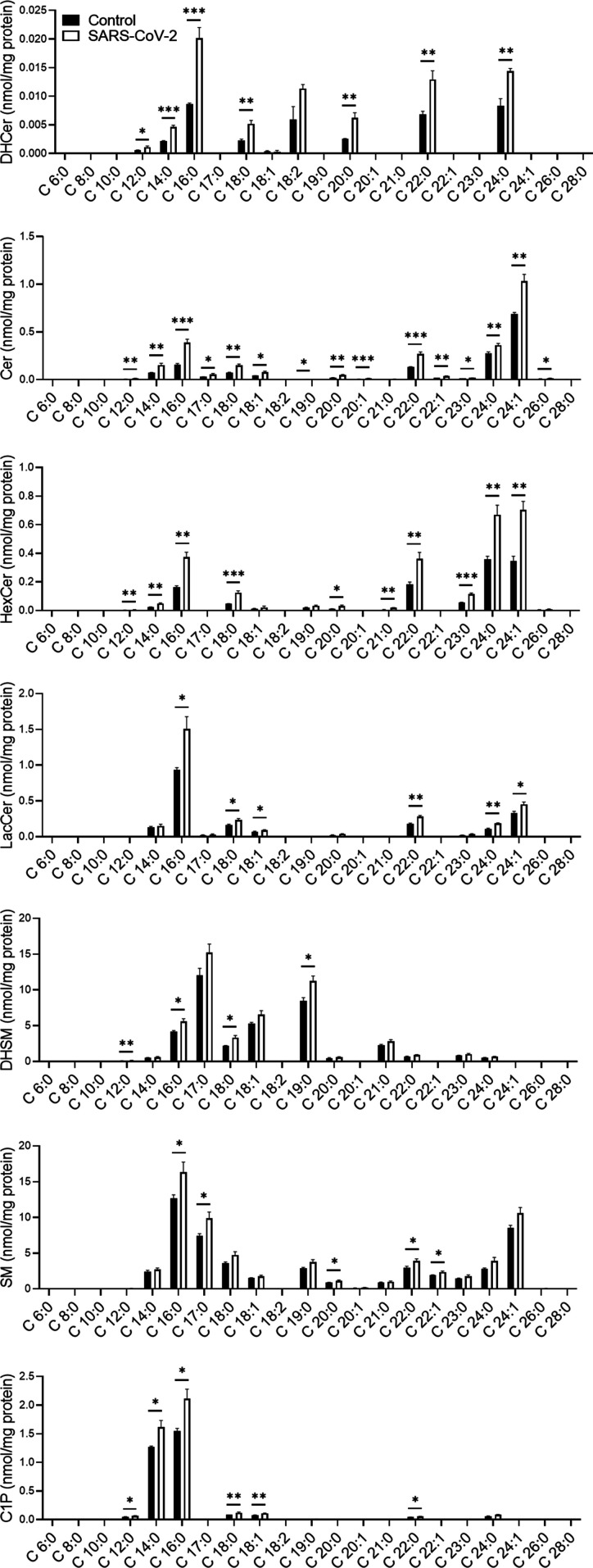
SARS-CoV-2 infection induces elevation of GSL levels in vitro. Vero E6 cells were infected with SARS-CoV-2 at a MOI of 5, and SL levels were quantified at 3 hpi by LC–MS. DHCer, dihydroceramide; Cer, ceramide; HexCer, hexosylceramide; LacCer, lactosylceramide; DHSM, dihydrosphingomyelin; SM, sphingomyelin; C1P, ceramide-1-phosphate. Data are the means of four biological replicates ± SEMs. Statistical analysis was performed using a two-tailed unpaired *t* test. *P*-values are indicated by asterisks, as follows: *P < 0.05, **P < 0.01, and ***P < 0.001. Differences with a *P*-value of 0.05 or less were considered significant. Graphs were generated using GraphPad Prism software version 8.4.3. Complete dataset in Table S1.

Table S1 Complete mass spectrometry data from in vitro experiments. Related to Figs 1, 2, and 4.

**Figure 3.**
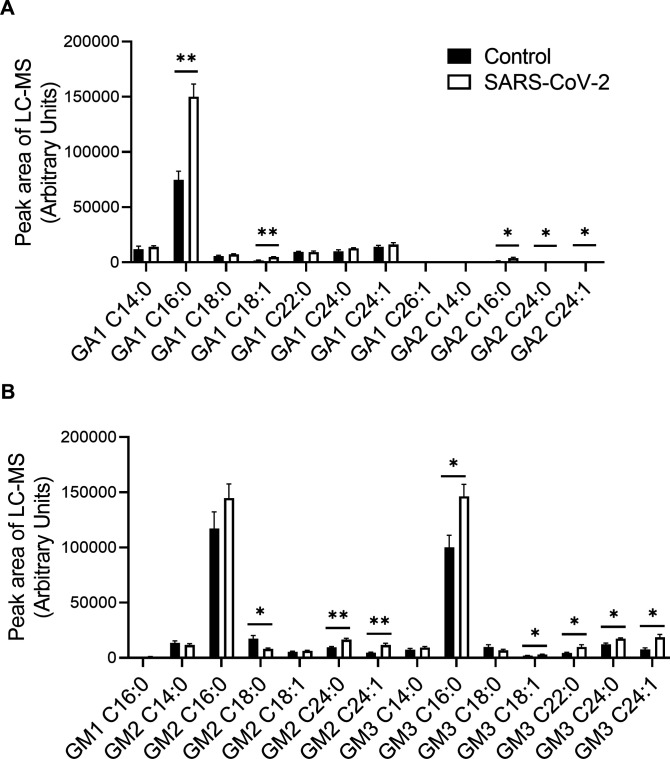
SARS-CoV-2 infection induces elevation of ganglioside levels in vitro. Vero E6 cells were infected with SARS-CoV-2 at a MOI of 5, and ganglioside levels were detected at 3 hpi by LC–MS. **(A)** Levels of asialogangliosides. **(B)** Levels of gangliosides. Data are the means of four biological replicates ± SEMs. The LC–MS peak area was divided by milligrams of protein in the sample for calibration. Statistical analysis was performed using a two-tailed unpaired *t* test. *P*-values are indicated by asterisks, as follows: *P < 0.05 and **P < 0.01. Differences with a *P*-value of 0.05 or less were considered significant. Graphs were generated using GraphPad Prism software version 8.4.3. Complete dataset in Table S2.

Table S2 Ganglioside mass spectrometry data. Related to Figs 3 and 7.

Table S3 Complete mass spectrometry data from in vivo experiments. Related to Figs 5 and 6.

Recent studies suggested a role of sphingolipids in modulating SARS-CoV-2 infection (Carpinteiro et al, 2020; Törnquist et al, 2021). In addition, we have recently shown that the synthesis of GSLs is necessary to support SARS-CoV-2 replication in vitro, suggesting alterations in SL levels upon SARS-CoV-2 infection (Vitner et al, 2021).

In this study, we show that SARS-CoV-2 induces an increase in the levels of SLs in vitro and in vivo and discuss the possible implications of such alterations.

## Results

### SARS-CoV-2 infection induces an increase in SL levels early upon infection

Previously reported data suggest that inhibiting GCS interrupts early stages in the replication cycle of SARS-CoV-2 (Vitner et al, 2021). Thus, SL levels were examined in Vero E6 cells at an early stage upon infection with SARS-CoV-2. At 3 hours post infection (hpi) with SARS-CoV-2, a significant elevation in 3-ketosphinganine (3KSa) (d16:0, d18:0, d18:1, and d20:0), sphinganine (Sa), sphingosine (So), and sphinganine-1-phosphate (d18:0-P and Sa1P) levels was detected (Fig 1).

The alteration of SL levels was also reflected by an elevation in the levels of many other downstream SLs; DHCer and Cer levels were significantly elevated in SARS-CoV-2-infected cells (Fig 2). In addition, SARS-CoV-2 induced a significant elevation in GSL levels and, to a lesser extent, in DHSM, SM and C1P levels (Fig 2). Hexosylceramide (HexCer, β-galactosylceramide [GalCer] and β-GlcCer) levels were mostly elevated, as well as the levels of lactosylceramides (LacCers) (Fig 2). The elevation was not specific to certain fatty acid chain lengths and seemed to reflect the distribution of the different species in Vero E6 cells (Fig 2). LacCer is the precursor of gangliosides, a family of sialic acid-containing GSLs, as well as of asialo-series gangliosides (Yu et al, 2011). Infection of E6 cells with SARS-CoV-2 induced elevation in the levels of the asialo-gangliosides GA1 and GA2 (Fig 3A) and the gangliosides GM2 and GM3 (Fig 3B). These data indicate elevation in the levels of many SLs, including GSLs and gangliosides, early after infection with SARS-CoV-2.

### A GCS inhibitor prevented the elevation in GSL levels upon SARS-CoV-2 infection

**Figure 4.**
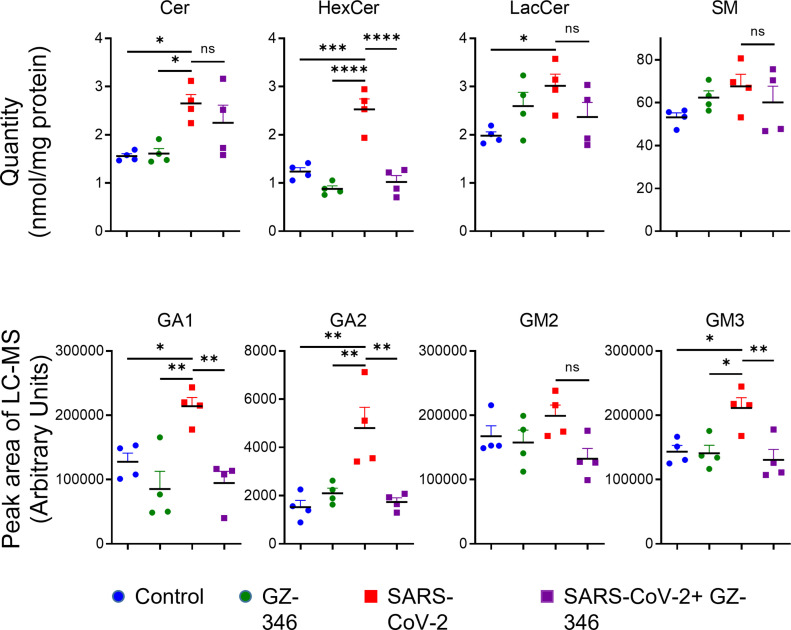
Treatment with GZ-346 prevents the elevation in the levels of HexCer and gangliosides upon SARS-CoV-2 infection in vitro. Vero E6 cells were infected with SARS-CoV-2 at a MOI of 5 with or without GZ-346 (10 μM). GZ-346 was added to the medium 1 h before infection. Cer, ceramide; HexCer, hexosylceramide; LacCer, lactosylceramide; SM, sphingomyelin; GA1, asialo GM1; GA2, asialo GM2. Data are the means of four biological replicates ± SEMs. Statistical analysis was performed using one-way ANOVA followed by Tukey’s multiple comparison test. *P*-values are indicated by asterisks, as follows: *P < 0.05, **P < 0.01, ***P < 0.001, and ****P < 0.0001. Differences with a *P*-value of 0.05 or less were considered significant. Graphs and analysis were performed using GraphPad Prism software version 8.4.3. Complete dataset in Table S1.

We have recently shown that GCS inhibitors disrupt the early stages of SARS-CoV-2 replication (Vitner et al, 2021). The antiviral effect of GCS inhibitors could be due to decreased levels of GSLs or elevated levels of ceramide. To determine which mechanism was applied, we examined the influence of a GCS inhibitor on the SL profile upon SARS-CoV-2 infection. The GCS inhibitor that was examined was (1R,2R)-nonanoic acid [2-(2′,3′-dihydro-benzo [1,4] dioxin-6′-yl)-2-hydroxy-1-pyrrolidin-1-ylmethyl-ethyl]-amide-l-tartaric acid salt (Genz-123346), termed hereafter GZ-346. GZ-346 is an analog of the FDA-approved drug eliglustat, which is indicated for the long-term treatment of adult patients with Gaucher disease type 1 (GD1) (Zhao et al, 2007). GZ-346 significantly prevented the elevation in the levels of HexCer, GA1, GA2, and GM3 upon SARS-CoV-2 infection (Fig 4). However, no significant differences in the levels of Cer, LacCer, SM, and GM2 were detected (Fig 4). Our data suggest that the induction of GSL biosynthesis by SARS-CoV-2 early upon infection is necessary for viral replication.

### SARS-CoV-2 infection induces elevation in the levels of SLs in mouse sera

**Figure S2.**
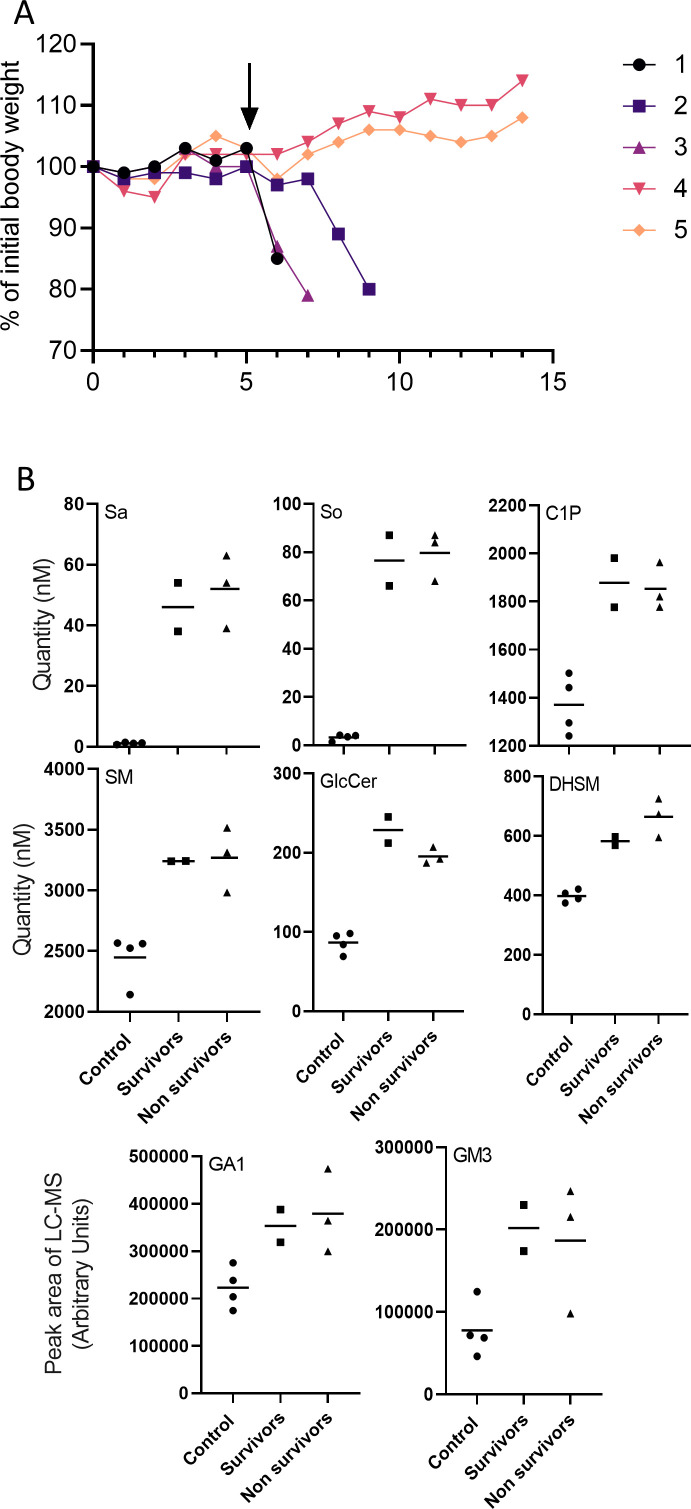
Sphingolipid levels in serum of SARS-CoV-2 infected mice. **(A)** Body weight (% of infection day 0) of five K18-hACE2 mice infected with SARS-CoV-2 (20 pfu, intranasal). Serum for sphingolipid analysis was taken at 5 dpi (marked by arrow). Mice 1–3 succumbed to death (nonsurvivors). Mice 4–5 survived (survivors). **(B)** Detailed presentation of selected sphingolipid levels divided into two subgroups of infected mice: survivors and nonsurvivors. Sa, sphinganine; So, sphingosine; C1P, ceramide-1-phosphate; DHSM, dihydrosphingomyelin; SM, sphingomyelin; HexCer, hexosylceramide; GA1, asialo GM1.

**Figure 5.**
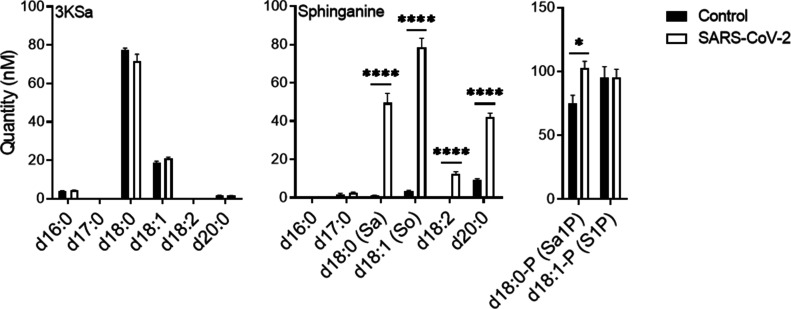
SARS-CoV-2 infection induces elevation in sphinganine levels in murine serum. K18-hACE2 transgenic mice were infected with SARS-CoV-2 (20 pfu, intranasally inoculated, n = 5) or uninfected (control, n = 4). Sphingoid base levels in serum samples obtained at day 5 post infection were analyzed by LC–MS. 3KSa, 3-ketosphinganine; Sa, sphinganine; So, sphingosine; Sa1P, sphinganine-1-phosphate; S1P, sphingosine-1-phosphate. Data are means± SEMs. Statistical analysis was performed using a two-tailed unpaired *t* test. *P*-values are indicated by asterisks, as follows: *P < 0.05, and ****P < 0.0001. Differences with a *P*-value of 0.05 or less were considered significant. Graphs were generated using GraphPad Prism software version 8.4.3. Complete dataset in Table S3.

Next, we examined whether the elevation in the levels of SLs induced by SARS-CoV-2 was also applied in vivo. Alterations in SL levels are implicated in the pathogenesis of various diseases, including lysosomal storage diseases, cardiovascular diseases, and neurodegenerative disorders (Platt, 2014; Borodzicz et al, 2015; Alessenko & Albi, 2020), and might have implications in SARS-CoV-2 infection pathology. SL levels were measured in serum 5 days post infection (dpi) of K18-hACE2-transgenic mice with SARS-CoV-2 just before the appearance of symptoms (Fig S2A). Five mice were analyzed: two asymptomatic mice that survived the infection and three symptomatic mice that succumbed to death (Fig S2A). Alterations in SL levels were detected in all mice. A marked increase in the levels of Sa, So, sphinganine d18:2, sphinganine d20:0, and Sa1P (44-, 24-, 42-, 5-, and 1.4-fold increases, respectively) were detected in the serum of SARS-CoV-2-infected mice (Figs 5 and S2B), suggesting significant activation of the SL biosynthesis pathway by SARS-CoV-2 in vivo.

**Figure 6.**
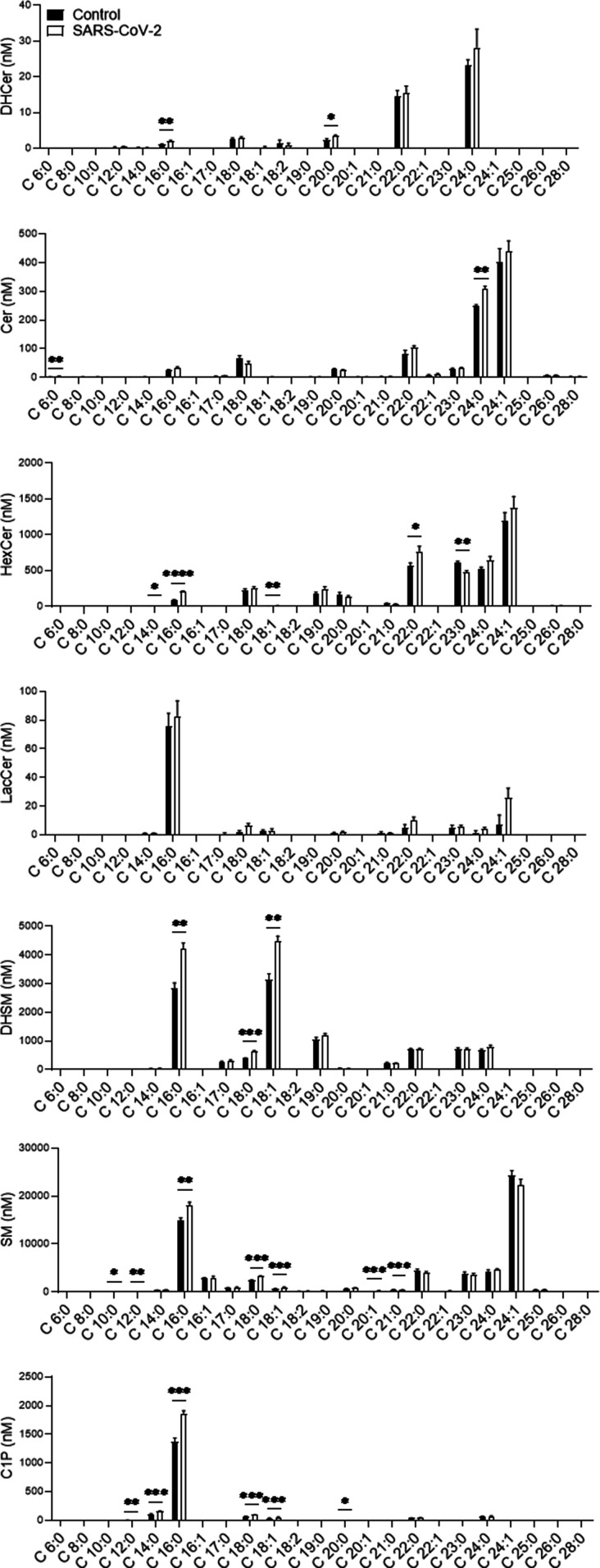
SARS-CoV-2 infection induces elevation in sphingolipid levels in murine serum. K18-hACE2 transgenic mice were infected with SARS-CoV-2 (20 pfu, intranasally inoculated, n = 5) or uninfected (control, n = 4). Sphingolipid levels in serum samples obtained at day 5 post infection were analyzed by LC–MS. DHCer, dihydroceramide; Cer, ceramide; HexCer, hexosylceramide; LacCer, lactosylceramide; DHSM, dihydrosphingomyelin; SM, sphingomyelin; C1P, ceramide-1-phosphate. Data are means± SEMs. Statistical analysis was performed using a two-tailed unpaired *t* test. *P*-values are indicated by asterisks, as follows: *P < 0.05, **P < 0.01, ***P < 0.001, and ****P < 0.0001. Differences with a *P*-value of 0.05 or less were considered significant. Graphs were generated using GraphPad Prism software version 8.4.3. Complete dataset in Table S3.

In addition to those for Sa, alterations in downstream SL levels were also detected: DHCer (C16:0 and C:20), Cer (C6:0 and C24:0), HexCer (C14:0, C16:0, C18:1, C22:0, and C23:0), DHSM (C16:0, C18:0, and C18:1), SM (C10:0, C12:0, C16:0, C18:0, C18:1, C20:1, and C21:0), and C1P (C12:0, C14:0, C16:0, C18:0, C18:1, and C20:0) levels were elevated in the sera of SARS-CoV-2–infected mice (Figs 6 and S2B). The levels of LacCer were unaltered (Fig 6).

**Figure 7.**
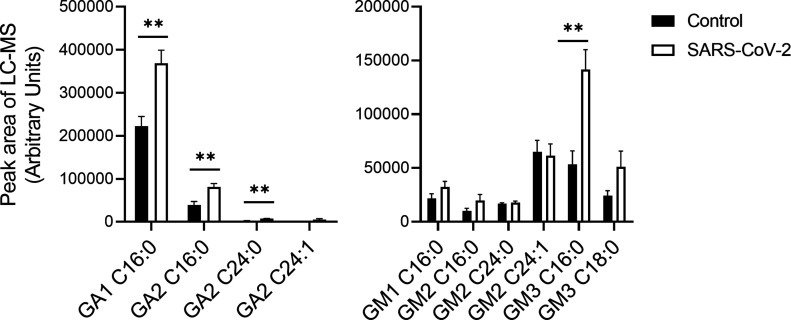
SARS-CoV-2 infection induces elevation in ganglioside levels in murine serum. K18-hACE2 transgenic mice were infected with SARS-CoV-2 (20 pfu, intranasally inoculated, n = 5) or uninfected (control, n = 4). Ganglioside levels in serum samples obtained at day 5 post infection were analyzed by LC–MS. The LC–MS peak area was divided by milligrams of protein in the sample for calibration. GA1, asialo GM1; GA2, asialo GM2. Statistical analysis was performed using a two-tailed unpaired *t* test. *P*-values are indicated by asterisks, as follows: **P < 0.01. Differences with a *P*-value of 0.05 or less were considered significant. Graphs were generated using GraphPad Prism software version 8.4.3. Complete dataset in Table S2.

In addition, similar to the elevation observed in infected cells (Fig 3), the levels of GA1 (C:16), GA2 (C16:0 and C24:0), and GM3 (C:16:0) were significantly increased in the sera of SARS-CoV-2–infected mice (Figs 7 and S2B).

## Discussion

SARS-CoV-2 infection induces elevation of SLs not only in cells but also in vivo. Levels of Sa, So, GA1, and GM3 were significantly increased in both cells and the murine model upon SARS-CoV-2 infection. The mechanism by which SARS-CoV-2 induces elevation in SLs needs to be elucidated. It was recently shown that pseudoviral SARS-CoV-2 induces acid sphingomyelinase activity, which affects the levels of ceramide (Carpinteiro et al, 2020). However, our data, demonstrating elevation of several SL species, together with previous data showing that inhibition of GCS is important to viral replication, suggest that the activity of an early enzyme in the SL synthesis pathway is elevated. These data are consistent with previous studies demonstrating the involvement of fatty acid synthase (FASN) in the replication of many viruses (Li et al, 2004; Kapadia & Chisari, 2005; Machesky et al, 2008; Yang et al, 2008; Diamond et al, 2010; Heaton et al, 2010; Perera et al, 2012; Huang et al, 2013; Tongluan et al, 2017; Chotiwan et al, 2018). Although the focus of this study is SLs, it is reasonable that other lipid levels are also altered by SARS-CoV-2.

SARS-CoV-2 significantly increased the levels of Sa and So in the serum. Alterations in So levels in symptomatic COVID-19 patients were recently described (Janneh et al, 2021). However, whereas symptomatic COVID-19 patients exhibited a robust decrease in their serum sphingosine levels, the mice exhibited the opposite effect. This difference might be a result of the time point of serum detection, and more studies should be conducted to answer this question.

Increases in the serum levels of Sa and So have implications in cardiovascular dysfunction; fumonisin B1 (FB1), a mycotoxin, has been shown to lead to altered SL biosynthesis and dose-dependent increases in serum and tissue Sa and So concentrations (Riley et al, 1993). In pigs, ingestion of FB1 has been shown to affect the cardiovascular system, causing cardiovascular dysfunction (Smith et al, 1996; Smith et al, 1999; Constable et al, 2000). Moreover, SL metabolism has been suggested to be involved in the pathophysiology of Kawasaki disease (KD), an acute systemic vasculitis (Konno et al, 2015). Inhibition of sphingolipid de novo synthesis has been shown to improve atherogenesis signs (Park et al, 2004; Hojjati et al, 2005; Glaros et al, 2007; Hornemann & Worgall, 2013; Borodzicz et al, 2015). An association between COVID-19 cardiovascular disease and KD has been reported (Viner & Whittaker, 2020). Preexisting cardiovascular disease seems to be linked with worse outcomes and increased risk of death in patients with COVID-19, whereas COVID-19 itself can also induce myocardial injury, arrhythmia, acute coronary syndrome and venous thromboembolism (Nishiga et al, 2020). Whether elevation of SL levels contributes to cardiovascular manifestations observed in SARS-CoV-2 has not been examined.

The elevation of gangliosides in response to SARS-CoV-2 is intriguing. Gangliosides are ubiquitously found in tissues and body fluids and are most abundantly expressed in the nervous system (Sipione et al, 2020). Anti-ganglioside antibodies (AGAs) are associated with an autoimmune condition in which the host’s immune system attacks the gangliosides of neurons (Willison et al, 2016). There is growing evidence indicating that neurological manifestations occur in patients with COVID-19 (Sharifian-Dorche et al, 2020; Andalib et al, 2021; Shehata et al, 2021). Our data showing increased levels of gangliosides in the sera of SARS-CoV-2–infected mice can provide a distinct potential mechanism by which elevated levels of host GSLs upon viral infection may trigger AGA generation.

GM1 is the most common AGA found in patients with GBS, whereas GQ1b is associated with MFS. Aside from one case report, a review of available articles yields no reported cases of COVID-19-related GBS or MFS that included positive tests for GM1 or GQ1b (Dufour et al, 2021). Our data indicate no elevation in GM1 levels in cells or sera upon SARS-CoV-2 infection, but GA1, GA2, and mostly GM3 levels were elevated (Figs 3 and 7). Interestingly, GM3 is a major ganglioside in the lungs (Iwamori et al, 1984) that is primarily infected by SARS-CoV-2. However, it is important to note that our study was performed in a transgenic mouse model and in Vero E6 cells. The specific models that were examined might affect the GSL profile that is being altered. Thus, a comprehensive unlimited analysis of gangliosides and anti-GSL antibodies in patients with COVID-19 might reveal novel target(s).

Interestingly, the SL elevation observed in the asymptomatic mice was similar to that observed in symptomatic mice. Thus, the elevation of SLs does not seem to be involved directly in pathogenesis and disease severity. However, if elevated SL indeed contributes to long-term manifestations, our data suggest that SL-related complications might also be present in asymptomatic individuals upon infection with SARS-CoV-2. This can suggest an explanation for signs, such as neurological and cardiovascular signs, with unknown etiology.

In addition to their potential role in pathology, SLs were found to be significantly useful markers of disease prediction, diagnosis, prognosis and treatment monitoring (Matanes et al, 2019). SLs have been linked to the pathophysiology of many diseases in the human body, including cardiovascular diseases, cancer, metabolic disorders, dementia, and mental diseases (Matanes et al, 2019) and recently also in COVID-19 (Janneh et al, 2021). Our data, showing elevation in SL levels in all infected mice, suggest the possible use of SLs as diagnostic biomarkers for viral diseases. This possibility should be further evaluated in patients rather than in mice.

Our data support the need for further research on the role of SLs in SARS-CoV-2 infection. First, SL quantification in patient serum will delineate whether the enrichment observed in the transgenic mouse model recapitulates the enrichment in humans. In addition, quantification of SLs upon other viral infections is needed to determine whether alterations in SL levels are common to many viruses. Next, studies exploring the role of SLs in cardiovascular and neurological complications in COVID-19 patients might open new therapeutic targets. The availability of FDA-approved drugs with the capacity to restore the elevation of serum GSL levels may reveal new strategies to prevent COVID-19 clinical complications.

## Materials and Methods

### Cell sample preparation

Vero E6 (ATCC CRL-1586) cells were obtained from the American Type Culture Collection. Cells were grown in DMEM supplemented with 10% FBS, MEM nonessential amino acids (NEAAs), 2 mM L-glutamine, 100 units/ml penicillin, 0.1 mg/ml streptomycin, and 12.5 units/ml nystatin (P/S/N) (Biological Industries). Cells were cultured at 37°C in a 5% CO_2_ and 95% air atmosphere.

Vero E6 cells were seeded at a density of 1 × 10^6^ cells per 60 mm plate. After incubating overnight, cells were treated in four replicates with 10 μM GZ-346. The cells were infected 1 h later with SARS-CoV-2 (MOI: 5). At 3 hpi, the cells were washed three times in cold PBS and collected with a rubber policeman.

GZ-346 ((1R,2R)-nonanoic acid [2-(2′,3′-dihydro-benzo [1,4] dioxin-6′-yl)-2-hydroxy-1-pyrrolidin-1-ylmethyl-ethyl]-amide-l-tartaric acid salt) was obtained from Sanofi. The compound was stored as a 5 mM stock solution in PBS at −20°C until use.

### Animal experiment

Treatment of animals was in accordance with regulations outlined in the U.S. Department of Agriculture (USDA) Animal Welfare Act and the conditions specified in the Guide for Care and Use of Laboratory Animals (National Institutes of Health, 2011). Animal studies were approved by the local ethics committee on animal experiments (protocol number M-51-20). Male and female K18-hACE2 transgenic (B6.CgTg(K18ACE2)2Prlmn/J HEMI) mice (Jackson Laboratories) were maintained at 20–22°C and a relative humidity of 50 ± 10% on a 12-h light/dark cycle, fed commercial rodent chow (Koffolk Inc.), and provided with tap water ad libitum. Animals were 6- to 8-wk old. All animal experiments involving SARS-CoV-2 were conducted in a BSL3 facility. Infection experiments were carried out using the SARS-CoV-2 isolate Human 2019-nCoV ex China strain BavPat1/2020, which was kindly provided by Prof. Christian Drosten (Charité) through the European Virus Archive—Global (EVAg Ref-SKU: 026V-03883). The original viral isolate was amplified by five passages, quantified by a plaque titration assay in Vero E6 cells, and stored at −80°C until use. The viral stock DNA sequence and coding capacity were confirmed as recently reported (Finkel et al, 2021). The SARS-CoV-2 BavPat1/2020 virus (20 pfu) diluted in PBS supplemented with 2% FBS (Biological Industries) was used to infect animals by intranasal instillation (20 μl) of anesthetized mice. Control groups were administered PBS. Serum samples were collected at day 5 post infection from SARS-CoV-2–infected and control mice. All sera were heat-inactivated (HI) (at 56°C for 30 min).

### Sphingolipid quantification

#### Sample preparation

Each cell pellet and each 50 μl serum sample were suspended in 100 μl of methanol/chloroform (1:1), and the samples were sent to The Metabolomics Innovation Centre (TMIC) for analysis. Each sample was mixed with 100 μl of a mixture of 5 deuterium-labeled sphingolipids as internal standards and 300 μl of methanol/chloroform (3:1) containing BHT as an antioxidant. The mixture was vortexed for 2 min at 1,000*g* and then ultrasonicated in an ice-water bath for 5 min before centrifugal clarification for 10 min at 21,000*g*. The clarified supernatant was collected for LC-MRM/MS, and the protein pellet was used to perform protein quantitation using a standardized Bradford procedure.

#### Calibration solutions and LC–MS

A mixed standard-substance stock solution of targeted sphingolipids was prepared at a concentration of 40 μM for each compound in methanol-chloroform (3:1) containing the same internal standards. This solution was serially diluted 1:4 (vol/vol) with the same solvent to obtain 10 calibration solutions. 10-μl aliquots of the calibration solutions and the sample solutions were injected onto an LC column (C8, 2.1 × 50 mm, 1.7 μm) to perform UPLC-MS/MS on a Waters Acquity UPLC system coupled to a 4000 QTRAP mass spectrometer, which was operated in multiple-reaction monitoring (MRM) mode with positive ion detection for sphingolipids and negative ion detection for sphingolipid phosphates. The mobile phase was 0.01% formic acid in water and acetonitrile-isopropanol (2:1) for binary-solvent gradient elution (25–100% organic solvent in 12.5 min), followed by a 3-min column cleanup and 4-min column equilibration at 400 μl/min and 55°C. The ion transitions for MRM detection of each sphingolipid were optimized by direction infusion of an individual standard solution to produce two ion transitions per compound. The UPLC-MRM/MS data files were recorded using Sciex Analyst 1.6 software and were processed using Sciex MultiQuant 2.0 software. Linear regression calibration curves of individual sphingolipids were constructed with internal-standard calibration, and the concentrations of sphingolipids detected in each sample were calculated from the calibration curves with the measured analyte-to-internal standard peak area ratios.

Gangliosides were detected on an LTQ-Orbitrap Velos Pro with high mass resolution detection (FWHM 60,000 at m/z 400) in a mass range of m/z 300–2,000 and in positive ion mode. Gangliosides were assigned based on comparison of the measured accurate masses of gangliosides to their theoretically calculated masses within an allowable mass error of 3 ppm and with the aid of standard substances of the gangliosides GM1, GM2, and GM3. The ion chromatograms of detected gangliosides were extracted using their accurate masses within a mass window of 3 ppm, and the peak areas were used for relative quantitation. Peak area was normalized to milligrams of protein.

### Statistical analysis

Statistical analyses were performed with a two-tailed unpaired *t* test or one-way ANOVA followed by Tukey’s multiple comparison test, as indicated in the figure legends. *P*-values are indicated by asterisks in the figures, as follows: *P < 0.05, **P < 0.01, ***P < 0.001, and ****P < 0.0001. Differences with a *P*-value of 0.05 or less were considered significant. The exact value of n is indicated in the figure legends. Data for all measurements are expressed as the means ± SEMs. Analyses were performed using GraphPad Prism software version 8.4.3.

## Data Availability

This manuscript does not have large-scale data sets to deposit to the public databases.

## Supplementary Material

Reviewer comments
